# Identification of HIV-1 sequences targeted by the miRNA Induced silencing complex (miRISC) using Argonaute 2 cross-linking and immunoprecipitation (HITS-CLIP)

**DOI:** 10.1186/1742-4690-10-S1-P33

**Published:** 2013-09-19

**Authors:** Agathe Eckenfelder, Natalia Pinzon Restrepo, Damien Ulveling, Jean-François Zagury, Hervé Seitz, Stéphane Emiliani, Sarah Gallois-Montbrun

**Affiliations:** 1 Inserm, U1016, Institut Cochin, Paris, France; 2 CNRS, UMR8104, Université Paris Descartes, Paris, France; 3 CNRS UPR 1142, IGH, Montpellier, France; 4 CNAM, Paris, France

## Background

The RNA interference pathway (RNAi) regulates nearly 50 percent of human transcripts at a post-transcriptional level. The effector complex of the pathway is the microRNA-induced silencing complex (miRISC) composed of one of the four Argonaute proteins (Ago1 to 4) associated with a miRNA. Once loaded into the miRISC, these small non-coding RNA guide the complex to specific mRNA sequences and the binding of the Ago protein to the transcripts can result either in its degradation or in translational repression. Several studies have shown that the miRNA pathway can also participate in the regulation of HIV-1 replication and latency. However, the molecular mechanisms involved remain to be explored. Using a HITS-CLIP (high throughput sequencing of RNA isolated by cross-linking and immunoprecipitation) approach, we identified viral RNA sequences targeted by the miRISC.

## Materials and methods

Results from our lab and other groups have shown that viral RNA was specifically co-immunoprecipitated (co-IP) with Argonaute proteins such as Ago2. We used an Ago2 CLIP strategy to trap and immunoprecipitate the miRNA:Ago2 :mRNA complexes. High throughput sequencing and bioinformatics analysis led to the identification of the viral RNA sequences targeted by the miRISC in the context of infection.

## Results

We performed 4 independent CLIP experiments in 293T cells overexpressing GFP-Ago2 and infected with HIV-1. Deep sequencing of small RNA in cell extracts before IP (input) were also done. From the 4 experiments, 70 million raw reads were obtained for the CLIP and 90 million for the small RNA input. These reads were aligned either with the human or with the viral genome. HIV-1 specific reads represent between 0,4 and 1% of total reads in the input and between 3 and 5% of total reads in the CLIP experiments. Computational analyses of these data showed around 50 clusters of reads of 60 to 200 nucleotides length, distributed over the viral genome. 32 of theses peaks are specifically enriched in the IP over the input and are likely to represent Ago2 binding sites. Some of them were previously identified, in particular in the nef region, but we also identified new regions that are likely to be the targets of Ago2 and that were not predicted by bioinformatics analysis.

## Conclusion

Data generated by our CLIP experiments allowed us to identify viral mRNA sequences that are specifically recruited to miRISC during viral infection. Our ongoing work is to characterize the role of Ago2 binding on these sequences during HIV-1 replication and define more precisely how the RNAi pathway is involved in the regulation of the virus.

